# Increasing metformin concentrations and its excretion in both rat and porcine ex vivo normothermic kidney perfusion model

**DOI:** 10.1136/bmjdrc-2019-000816

**Published:** 2020-08-17

**Authors:** Rene A Posma, Leonie H Venema, Tobias M Huijink, Andrie C Westerkamp, A Mireille A Wessels, Nynke J De Vries, Frank Doesburg, J Roggeveld, Petra J Ottens, Daan J Touw, Maarten W Nijsten, Henri G D Leuvenink

**Affiliations:** 1 Department of Critical Care, University of Groningen, University Medical Center Groningen, Groningen, The Netherlands; 2 Department of Surgery, University of Groningen, University Medical Center Groningen, Groningen, The Netherlands; 3 Department of Clinical Pharmacy and Pharmacology, University of Groningen, University Medical Center Groningen, Groningen, The Netherlands

**Keywords:** metformin, transport, pharmacokinetics, lactic acidosis

## Abstract

**Introduction:**

Metformin can accumulate and cause lactic acidosis in patients with renal insufficiency. Metformin is known to inhibit mitochondria, while renal secretion of the drug by proximal tubules indirectly requires energy. We investigated whether addition of metformin before or during ex vivo isolated normothermic machine perfusion (NMP) of porcine and rat kidneys affects its elimination.

**Research design and methods:**

First, Lewis rats were pretreated with metformin or saline the day before nephrectomy. Subsequently, NMP of the kidney was performed for 90 min. Metformin was added to the perfusion fluid in one of three different concentrations (none, 30 mg/L or 300 mg/L). Second, metformin was added in increasing doses to the perfusion fluid during 4 hours of NMP of porcine kidneys. Metformin concentration was determined in the perfusion fluid and urine by liquid chromatography-tandem mass spectrometry.

**Results:**

Metformin clearance was approximately 4–5 times higher than creatinine clearance in both models, underscoring secretion of the drug. Metformin clearance at the end of NMP in rat kidneys perfused with 30 mg/L was lower than in metformin pretreated rats without the addition of metformin during perfusion (both p≤0.05), but kidneys perfused with 300 mg/L trended toward lower metformin clearance (p=0.06). Creatinine clearance was not different between treatment groups. During NMP of porcine kidneys, metformin clearance peaked at 90 min of NMP (18.2±13.7 mL/min/100 g). Thereafter, metformin clearance declined, while creatinine clearance remained stable. This observation can be explained by saturation of metformin transporters with a Michaelis-Menten constant (95% CI) of 23.0 (10.0 to 52.3) mg/L.

**Conclusions:**

Metformin was secreted during NMP of both rat and porcine kidneys. Excretion of metformin decreased under increasing concentrations of metformin, which might be explained by saturation of metformin transporters rather than a self-inhibitory effect. It remains unknown whether a self-inhibitory effect contributes to metformin accumulation in humans with longer exposure times.

**Significance of this study**

**What is already known about this subject?**

- Metformin can accumulate and cause lactic acidosis in patients with renal insufficiency, which is a severe complication of metformin therapy.
- Metformin is known to inhibit mitochondria, while renal secretion of the drug by proximal tubules indirectly requires energy and might be saturated.

**What are the new findings?**

- Metformin was secreted during normothermic machine perfusion of both rat and porcine kidneys.
- Metformin clearance was reduced under increasing concentrations of the drug, indicating saturation of transporters affecting its elimination.
- There is no indication that other tubular functions were altered when exposed to concentrations of metformin considered to be toxic in vivo.

**How might these results change the focus of research or clinical practice?**

- Further research is needed to elucidate the underlying mechanism how metformin-induced lactic acidosis develops.
- Is metformin secretion concentration-dependently reduced in patients using metformin?
- Does the severity of chronic kidney disease affect the threshold at which metformin transporters are saturated?

## Introduction

The biguanide metformin is widely used as an antihyperglycemic agent to treat patients with type 2 diabetes. Although the exact mechanism of action is unknown, it is generally presumed that mild inhibition of complex I within the mitochondrial electron transport underlies most of the pleiotropic effects of metformin.^1^ The primary mode of elimination is excretion of the unchanged drug in urine.^2^ In some patients with acute renal insufficiency, metformin can accumulate and cause lactic acidosis. The reported incidence of metformin-associated lactic acidosis ranges from 3 to 10 per 100 000 patient-years and is associated with a high mortality rate. However, the full clinical context or metformin blood concentration is often not reported, making it difficult to distinguish metformin-associated from metformin-induced lactic acidosis, respectively.^3^ Because of the risk to develop metformin-associated lactic acidosis (defined as lactate ≥5 mmol/L, pH <7.35 and metformin concentration >5 mg/L),^3^ metformin is currently contraindicated in patients with severe chronic kidney disease.^4^ Moreover, it is recommended to be used with caution when conditions are present that may reduce renal function,^4^ which in clinical practice leads to discontinuation of metformin 48 hours before surgery or coronary angiography.^5 6^

Renal clearance of metformin is approximately four times higher than the glomerular filtration rate, indicating secretion of the drug.^2^ In the proximal tubules, metformin is primarily taken up by organic cation transporter (OCT)-2 through facilitated transport down an electrochemical gradient.^7^ Subsequently, metformin is luminally excreted by OCT-1 and multidrug and toxin extrusion transporter (MATE)-1 and MATE-2.^2^ The latter are secondary active transporters with an ion-coupled substrate.^7^ Although indirect, the mediation of transcellular movement against gradients requires ATP.^7^ Abundant mitochondria in the epithelial cells of the proximal tubule, estimated as 30%–40% of the total volume, support the extraordinarily high energy demand of these cells.^8^ In times that MATE transporters were not yet characterized,^9^ accumulation of cimetidine in canine kidney cortex slices and of tetraethylammonium in rat kidney slices, respectively, was hampered when exposed to hypoxia or potassium cyanide, underscoring that organic cation transport in general is an energy-dependent process.^10 11^ However, it is unknown to which extent metformin transport specifically is influenced by changes in cellular energy.

Combining the paradigms that mitochondria in the proximal tubules facilitate transport with mild inhibition of mitochondria by metformin, we investigated whether the addition of metformin to two ex vivo isolated normothermic machine perfusion (NMP) rat and porcine kidney models affects the excretion of the drug.

## Research design and methods

### Rat kidney study

#### Animals

Male Lewis rats (weighing 270–300 g) were obtained from Harlan Laboratories (Boxmeer, The Netherlands). Animals received care according to the Dutch Law on Animal Experiments and had ad libitum access to food and water.

#### Experimental design

**Figure 1:**
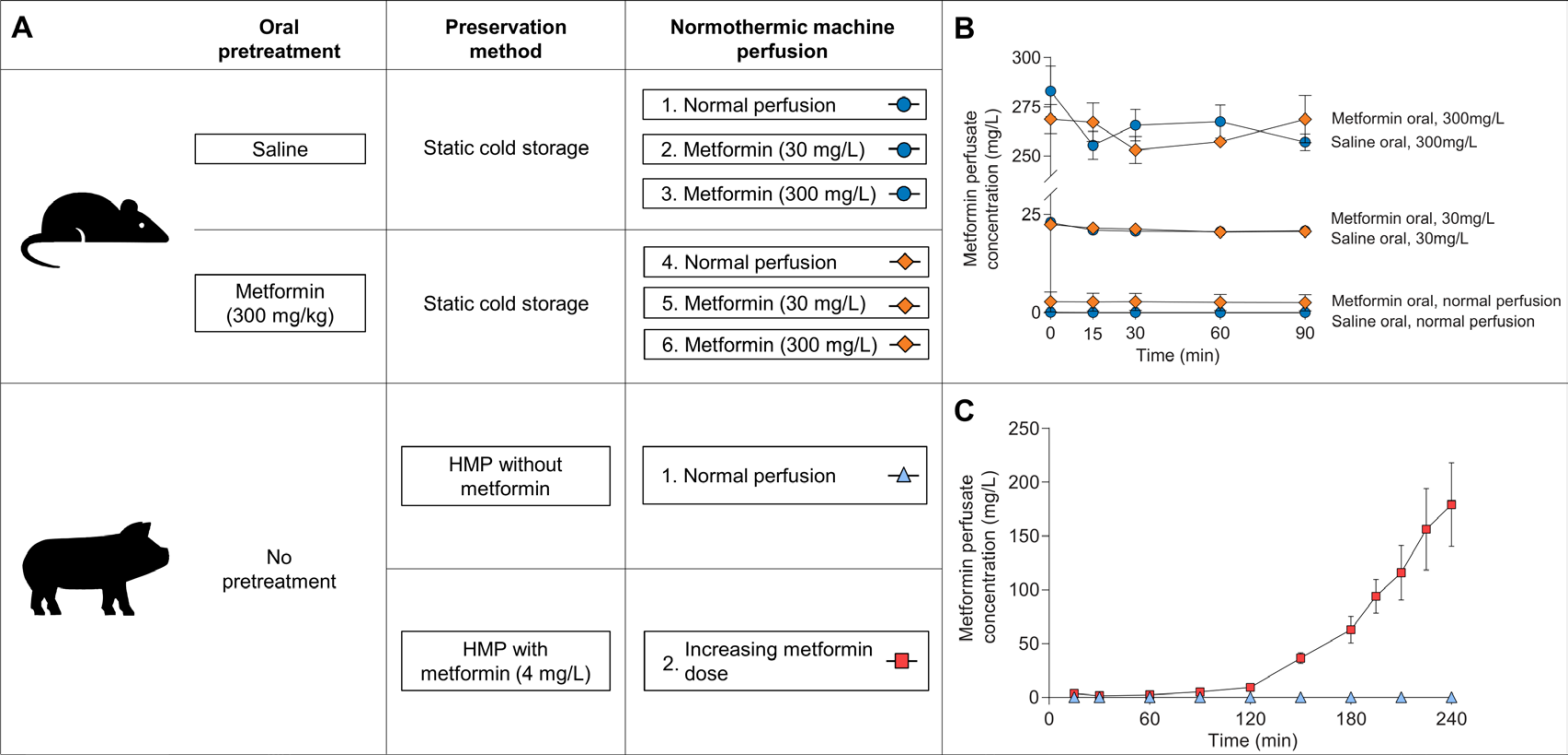
(A) Schematic representation of the experimental groups in the rat and porcine kidney study, respectively. Oral pretreatment of rats via oral gavage occurred twice the day before nephrectomy. Rat kidneys were preserved for 24 hours with static cold storage, while 3 hours of hypothermic machine perfusion (HMP) was performed for porcine kidneys. Normothermic machine perfusion in rat kidneys lasted 90 min, whereas porcine kidneys were perfused for 240 min. (B) Metformin concentration measured using liquid chromatography tandem-mass spectrometry in the perfusate during normothermic machine perfusion of rat kidneys. (C) Metformin concentration in the perfusate during normothermic machine perfusion of porcine kidneys.

Rats were treated with 300 mg/kg body weight metformin (1,1-dimethylbiguanide hydrochloride, Sigma-Aldrich, St. Louis, Missouri, USA) dissolved in saline or saline alone via oral gavage at 12 and 2 hours before nephrectomy. In previous experiments, this dose resulted in metformin concentrations comparable to those found in humans during maintenance metformin therapy.^12^ After nephrectomy, experimental kidneys were preserved by static cold storage for 24 hours, while the contralateral (ie, non-perfused) kidney was stored in some cases at −80°C for further analysis. Experimental kidneys were subsequently perfused for 90 min in an ex vivo NMP setup with the addition of no metformin, 30 mg/L metformin or 300 mg/L metformin. The latter metformin concentration was chosen to examine its effect on active transport processes, but should be considered to be massive as this concentration was only reported in an exceptional case of excessive metformin ingestion.^13^ The rat study had thus five metformin groups and one control group, all consisting of five to six kidneys (figure 1A).

#### Organ retrieval and preservation

Rats were anesthetized with 2%–5% isoflurane, and laparotomy was done via a midline incision. After anticoagulation with 250 IU heparin (Leo Pharma, Bellerup, Denmark), cardiac arrest was achieved by inducing a heart tamponade manually by external compression. After 15 min of warm ischemia time, nephrectomy of the left kidney was performed. The renal artery and ureter were cannulated. The renal vein was not cannulated. The kidney was flushed in situ with 10 mL saline and 5 mL 4°C Belzer University of Wisconsin (UW) Cold Storage Solution (Bridge to Life, Columbia, South Carolina, USA). Once removed, kidneys were flushed again with cold UW and stored in UW at 4°C for 24 hours.

#### Normothermic machine perfusion

The rat NMP methods were described previously.^14^ Briefly, a pressure-controlled NMP was performed using a roller pump (Ismatec ISM404, Zürich, Switzerland). The pressure was set at 102 mm Hg and measured at the renal artery using a pressure sensor (Edwards Lifesciences, Irvine, California, USA). The perfusion fluid consisted of 100 mL William’s Medium E supplemented with 30 mmol/L HEPES, 50 g/L albumin, and 7 mmol/L creatinine (all Sigma-Aldrich, St. Louis). According to the experimental treatment group, no metformin, 30 mg/L metformin or 300 mg/L metformin was added to the perfusion fluid at the start of NMP. Perfusion fluid was oxygenated with 95% oxygen and 5% carbon dioxide (carbogen) with a flow of 0.5 L/min. The temperature of the perfusion fluid was maintained stably at 37°C using a water bath and heat exchanger (Julabo, Seelbach, Germany).

### Porcine kidney study

#### Animals

Kidneys from female Dutch Landrace pigs were collected from a commercial abattoir after the animals were stunned and exsanguinated according to standard procedures. During exsanguination, 1 L of blood was obtained in a beaker containing 25 000 IU of unfractionated heparin (Leo Pharma).

#### Experimental design

The experimental group consisted of seven kidneys, in which 2 mg metformin (4 mg/L) was added at the start of 3 hours of hypothermic machine perfusion (HMP). Before the start of NMP, 3.3 mg (4 mg/L) metformin was added to the perfusion fluid. Subsequently during NMP, the infusion speed of an infusion pump containing metformin dissolved in Ringer’s lactate solution (in a concentration of 20 mg/mL) was increased every half hour according to a prespecified schedule presented in online supplementary table 2. This schedule was based on human pharmacokinetic data indicating that metformin clearance is four times higher than the creatinine clearance.^2^ In the control group, six kidneys were perfused without addition of metformin (figure 1A).

10.1136/bmjdrc-2019-000816.supp1 Supplementary data

#### Organ retrieval and preservation

After 30 min of warm ischemia time, the kidney was flushed with 180 mL cold saline. Subsequently, the kidney was preserved with oxygenated HMP (<7°C) using a commercially available pulsatile pressure-controlled pump system (Kidney Assist Transport, Organ Assist, Groningen, The Netherlands) for 3 hours. The kidneys were perfused with 500 mL UW machine perfusion solution (Belzers MP, Bridge to Life), supplemented with 2 mg metformin in the experimental group. The mean arterial pressure was set at 25 mm Hg. With a fixed flow of 100 mL/min, 100% oxygen was supplied to the oxygenator (Hilite LT 1000, Medos Medizintechnik, Stolberg, Germany).

#### Normothermic machine perfusion

A disposable perfusion circuit similar to that of HMP was used during NMP.^15^ Four hours of NMP was performed with 500 mL leukocyte depleted (BioR 02 plus, Fresenius Kabi, Bad Homburg, Germany) autologous oxygenated (carbogen, flow 0.5 L/min) blood diluted with 300 mL Ringer’s lactate solution, 10 mL glucose 5% (both Baxter, Utrecht, The Netherlands) and 10 mL sodium bicarbonate 8.4% (B. Braun, Melsungen, Germany). Before the start of NMP, the perfusion fluid was primed with 1000/200 mg amoxicillin-clavulanate (Sandoz, Almere, The Netherlands), 6 mg dexamethasone (B. Braun), 90 mg creatinine, 6 mg mannitol and 2 mg sodium nitroprusside (all Sigma-Aldrich; online supplementary table 1). In the experimental group, 3.3 mg metformin was added to the perfusion fluid before the start of NMP. During NMP, metformin was infused in the experimental group using an Alaris syringe pump (BD, Wokingham Berkshire, UK) controlled by a custom-made Android application to infuse according to a predefined schedule (online supplementary table 2). During NMP, in both groups a nutrient mixture was infused including amino acids, glucose, insulin and sodium bicarbonate (online supplementary table 1). Of the compounds added before and during NMP, nitroprusside was given as vasodilator to increase renal blood flow,^16^ mannitol was given to decrease cellular swelling,^17^ while leukocytes were filtered out of the blood and dexamethasone was administered, respectively, to decrease the inflammatory response.^18^ These compounds are proposed as preservation strategy surrounding kidney transplantation to reduce ischemia-reperfusion injury,^19^ and have been deemed necessary to support perfusion and renal function when establishing our NMP model (non-published data).

The system measured pressure just before the renal artery (Edwards Lifesciences) and was set to achieve a mean arterial pressure of 80 mm Hg. The arterial flow was recorded using an ultrasonic clamp-on flow probe (ME7PXL, Transonic Systems, Ithaca, New York, USA). The renal vein was not cannulated. Urine was collected and was replaced with a corresponding volume of Ringer’s lactate solution (Baxter). The temperature of the perfusion fluid was maintained at 37°C using an integrated heat exchanger of the oxygenator that was connected to a water bath (Julabo). The insulated perfusion box was heated using a radiator/ventilator combination (Tristar Europe, Tilburg, The Netherlands).

### Laboratory analysis

Perfusion fluid and urine samples collected during NMP were centrifuged (1300 g for 10 min in the rat model and 1000 g for 12 min in the porcine model, respectively, both at 4°C) and the supernatant was stored at −80°C for future analysis. After perfusion, cortical kidney tissue was immediately frozen in liquid nitrogen. Only during the last 30 min of NMP in the rat kidney study, sufficient urine was collected to determine metformin clearance. Because only three urine samples per pretreatment group of kidneys perfused with 300 mg/L metformin were available, both pretreatment groups were combined for statistical analyses.

#### Metformin concentration analysis

Metformin concentration was determined in whole blood, plasma, urine, and kidney tissue by liquid chromatography-tandem mass spectrometry (LC-MS/MS). In order to determine the metformin concentration in tissue, frozen cortical kidney tissue was crushed at liquid nitrogen temperature using a mortar and pestle to a fine powder. After a single freeze-thaw cycle, the mass approximated the consistency of whole blood and was deemed adequately homogenized to determine the metformin concentration. For all specimens, a volume of 10 µL sample was mixed with 750 µL precipitation reagent, consisting of a mixture of methanol and acetonitrile (4:21, vol/vol) and ^2^H_6_-metformin 0.1 mg/L (J.H. Ritmeester, Nieuwegein, The Netherlands). The samples were then centrifuged at 10 000 g for 5 min, and 5 µL of the supernatant was injected on the LC-MS/MS. The analysis was performed on a triple-stage quadrupole Quantum Access Max mass spectrometer coupled with a Vanquish ultra-performance liquid chromatography pump, a Vanquish autosampler and a Vanquish column oven (Thermo Scientific, San Jose, California, USA). Validation of the bioanalyses was performed according to the guidelines of the European Medicine Agency regarding validation of a bioanalysis method.^20^

Determination of creatinine and sodium in plasma and urine was performed by the clinical chemistry laboratory of the University Medical Center Groningen according to standard biochemical methods. Creatinine clearance, metformin clearance and fractional sodium excretion were calculated to estimate glomerular filtration rate, metformin excretion and tubular function, respectively (table 1). Clearance was calculated by dividing the elimination rate in urine from the previous timepoint at which urine was collected to the current timepoint by the current perfusion fluid concentration (table 1). Metformin-to-creatinine clearance ratio was calculated to determine the proportion of metformin that is eliminated more than creatinine, indicating secretion of the drug when assuming negligible creatinine secretion.

**Table 1:** Equations to calculate different parameters

Parameter (unit)	Equation
Clearance at, eg, 90 min after NMP (mL/min)	$\frac{Urine\;production_{t60\to t90}\cdot urine\;concentration_{t90}}{Perfusion\;fluid\;concentration_{t90}}$
Creatinine clearance (mL/min/100 g)	$\frac{\frac{Urine\;creatinine[\mu mol/L]\cdot urine\;production\;[mL/min]}{Perfusion\;fluid\;creatinine\;[\mu mol/L]}}{Kidney\;weight\;[100g]}$
Metformin clearance (mL/min/100 g)	$\frac{\frac{Urine\;metformin\;[mg/L]\cdot urine\;production\;[mL/min]}{Perfusion\;fluid\;metformin\;[mg/L]}}{Kidney\;weight\;[100g]}$
Metformin-to-creatinine clearance ratio	$\frac{Metformin\;clearance\;[mL/min/100g]}{Creatinine\;clearance\;[mL/min/100g]}$
Fractional sodium excretion (%)	$100\cdot \frac{Urine\;sodium\;[mmol/L]\cdot perfusion\;fluid\;creatinine\;[\mu mol/L]}{Perfusion\;fluid\;sodium\;[mmol/L]\cdot urine\;creatinine\;[\mu mol/L]}$
Oxygen consumption (mLO_2_/min/100 g)	$\frac{Flow\;[L/min]\cdot (arterial\;oxygen\;content\;[mLO_{2}/L]-venous\;oxygen\;content\;[mLO_{2}/L])}{Kidney\;weight[100g]}$
Oxygen content in porcine model (mLO_2_/L)	$24.794\left[\frac{mLO_{2}}{Hb\frac{mmol}{L}}at37℃\right]\cdot SO_{2}\left[fraction\right]\cdot hemoglobin\left[\frac{mmol}{L}\right]$ $+0.225\left[mLO_{2}/kPa\right]\cdot PO_{2}\left[kPa\right]$

Units of the parameters used within the equations are depicted with hard brackets to avoid confusion with regular brackets. In the porcine study, a total oxygen binding capacity of hemoglobin of 24.8 mLO_2_/mmol and 0.225 mLO_2_/kPa oxygen solubility in water at 37°C was assumed.

NMP, normothermic machine perfusion; PO_2_, arterial or venous partial pressure of oxygen; SO_2_, arterial or venous oxygen saturation.

#### Renal metabolic markers

Oxygen consumption was calculated based on the difference between the arterial and venous oxygen content (table 1).^18^ The total oxygen content in the rat kidney study was measured directly in the arterial and venous perfusion fluid (PreSens, Regensburg, Germany). In the porcine kidney study, arterial and venous samples were analyzed using an ABL800 blood gas analyzer (Radiometer, Brønhøj, Denmark). Subsequently, the oxygen content of the perfusion fluid was calculated by adding the free dissolved oxygen fraction to the hemoglobin bound oxygen fraction. We assumed a total oxygen binding capacity of hemoglobin of 24.8 mLO_2_/mmol and 0.225 mLO_2_/kPa oxygen solubility in water at 37°C (table 1).

Glucose and lactate levels in the rat study were measured using the same blood gas analyzer. Lactate production and glucose consumption throughout the rat kidney experiment were calculated by subtracting the lactate or glucose level at the start of NMP from the level at the end of the experiment. The addition of Ringer’s lactate to the perfusion fluid influenced our ability to determine lactate production in the porcine kidney study accurately. As an indicator of the energetic state of the kidney, ATP content was determined according to previously reported methods in porcine cortical tissue obtained at the end of NMP.^21^

#### Real-time quantitative PCR

Total RNA was extracted from kidney sections using TRIzol (Life Technologies, Gaithersburg, Maryland, USA). Samples were verified for the absence of genomic DNA contamination by performing real-time PCR reactions in which the addition of reverse transcriptase was omitted, using β-actin primers. For complementary DNA (cDNA) synthesis, 1 µL T11VN Oligo-dT (0.5 µg/µL) and 1 µg messenger RNA (mRNA) were incubated for 10 min at 70°C and subsequently cooled. cDNA was synthesized by adding a mixture containing 0.5 µL RNase water (Promega, Madison, Wisconsin, USA), 0.5 µL RnaseOUT Ribonuclease inhibitor, 4 µL 5 times first strand buffer, 2 µL dithiothreitol, 1 µL deoxyribonucleotide triphosphate and 1 µL M-MLV reverse transcriptase (all Invitrogen, Carlsbad, California, USA). The mixture was kept at 37°C for 50 min. Subsequently, reverse transcriptase was inactivated by incubating the mixture for 15 min at 70°C. Amplification of several gene fragments involved in renal tubular transport was done with the primer sets outlined in online supplementary table 3. cDNA obtained from rats was used to test primer efficiency and as an internal reference during PCR. Gene expression was normalized with the mean of β-actin mRNA content. PCR was carried out on the TaqMan 7900HT Real-Time PCR system (Applied Biosystems, Waltham, Massachusetts, USA), according to procedures outlined earlier.^22^ The 2^-ΔΔCt^ method was used to calculate the expression mRNA levels, where the Ct value represents the difference between cycle threshold values.

#### Statistical analysis

All data are expressed as mean±SEM. When comparing two groups at a single time point, differences were assessed using an unpaired Student’s t-test and, in case of more than two independent groups, using analysis of variance (ANOVA). To determine the total oxygen consumption, the area under the curve was approximated by dividing the curve into segments of 15 min, calculating the area of the trapezoids under these segments using Excel 2010 (Microsoft, Redmond, Washington, USA). Analyses were performed using SPSS Statistics V.23 (IBM, Armonk, New York, USA). A centered fifth-order polynomial model was used to fit the cross-sectional relation of metformin perfusate concentration with the urinary elimination rate (online supplementary figure 1a). Based on these results, non-linear pharmacokinetics using a Michaelis-Menten model were assessed and the corresponding Michaelis-Menten constant (K_m_) with 95% CI was calculated in Prism V.8.0.1 (GraphPad Software, San Diego, California, USA).

A linear mixed-effects repeated measures model with random slope and intercept was used to analyze the impact of the treatment (metformin or control) on fractional sodium excretion, oxygen consumption and pH over time. Fixed effects were time, treatment group and the interaction of treatment group with time. Among metformin-treated porcine kidneys, a similar analysis was performed using type of clearance (metformin or creatinine) instead of treatment group as fixed effect. Individual kidneys were considered as random effect. The covariance matrix of residuals used in all models was unstructured. A restricted maximum likelihood approach was used. Modeling was performed using Stata V.14.2 (StataCorp, College Station, Texas, USA). All statistical tests are two-tailed, and a p≤0.05 was considered to be statistically significant.

## Results

### Metformin perfusion fluid concentration

Metformin perfusion fluid concentrations in the rat kidney study were adequately achieved for each treatment group (figure 1B). Rats pretreated with metformin whose kidneys were subsequently perfused without the addition of metformin yielded a perfusion fluid metformin concentration of 2.8±2.6 mg/L at the start of NMP. Oral metformin or saline pretreatment of rats did not affect metformin perfusion fluid concentration at the start of NMP in the 30 mg/L group (22.4±0.1 (metformin pretreated) vs 23.0±0.5 (saline pretreated) mg/L, p=0.39) or in the 300 mg/L group (268.8±7.4 (metformin pretreated) vs 283.0±12.9 (saline pretreated) mg/L, p=0.37), respectively.

For the first 90 min during NMP in the porcine kidney study, metformin perfusion fluid concentration remained stable with a maximum of 5.1±1.2 mg/L (figure 1C). After that, metformin perfusion fluid concentration increased rapidly until a concentration of 179.3±38.8 mg/L was reached at the end of the experiment. No significant differences were found when comparing metformin concentrations measured in whole blood and plasma (online supplementary figure 2).

### Metformin clearance

**Figure 2:**
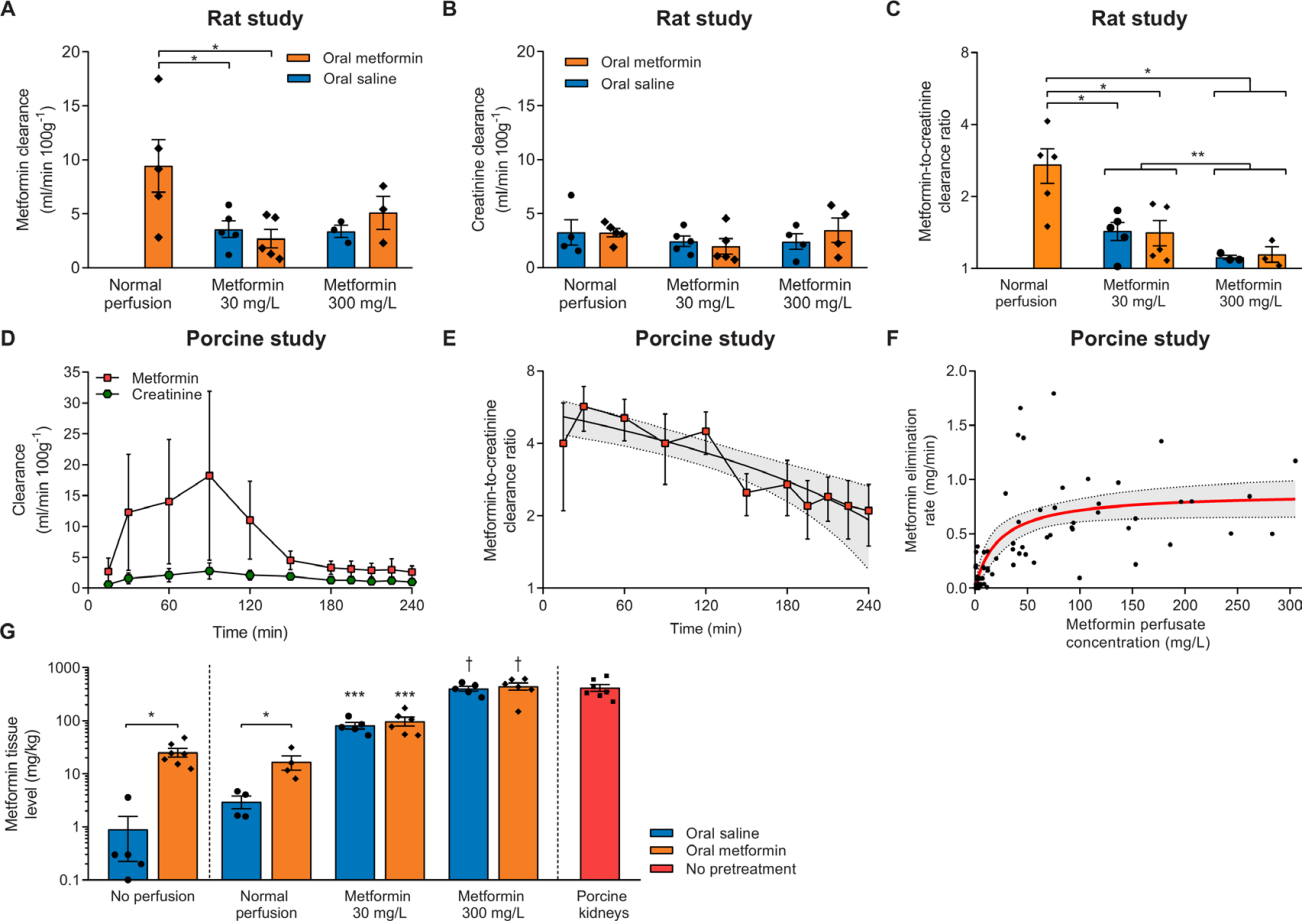
(A) Metformin clearance during the last 30 min of normothermic machine perfusion (NMP) of rat kidneys. Only three urine samples per pretreatment group in the 300 mg/L metformin group were available for determination of metformin concentration. *P<0.05. (B) Creatinine clearance during the last 30 min of NMP of rat kidneys. (C) Metformin-to-creatinine clearance ratio shown on a logarithmically transformed scale during the last 30 min of NMP of rat kidneys. Because only three urine samples per pretreatment group in the 300 mg/L metformin groups were available for the determination of the metformin concentration, both pretreatment groups were combined for statistical analysis. *P<0.05. **P<0.05 when combining pretreatment groups. (D) Metformin and creatinine clearance during NMP of metformin-treated porcine kidneys. (E) Metformin-to-creatinine clearance ratio of metformin-treated porcine kidneys displayed on a logarithmically transformed scale. Shown is a linear regression trend line with a 95% CI (dashed lines with the gray area in between). (F) Relation of metformin perfusate concentration with urinary elimination rate of metformin in the porcine kidney study. Displayed in red is a fit with 95% CI (dashed lines with the area in between) obtained from a Michaelis-Menten model. (G) Tissue metformin concentration in non-perfused rat kidneys, and after NMP in both the rat kidney study and porcine kidney study. *p<0.05. ***P<0.05 vs controls. †P<0.05 vs controls and kidneys perfused with 30 mg/L metformin irrespective of pretreatment. Except for panel F, data are expressed as mean±SEM.

Metformin clearance during the last 30 min of NMP in rat kidneys only pretreated with metformin was 2.7±0.4 times higher than creatinine clearance (figure 2A–C). Compared with rats that were pretreated with metformin and whose kidneys subsequently were perfused without the addition of metformin, metformin excretion in kidneys perfused with 30 mg/L metformin was decreased, irrespective of metformin pretreatment. Metformin clearance and the metformin-to-creatinine clearance ratio was even further decreased in kidneys perfused with 300 mg/L metformin, as compared with metformin pretreated rats that were subsequently perfused without addition of metformin, and when compared with kidneys perfused with 30 mg/L metformin when both pretreatment groups were combined (figure 2A–C). Of note, creatinine clearance did not differ during the last 30 min of NMP in the rat study (figure 2B). Non-linear curve fitting of the relation of metformin perfusate concentration with urinary elimination rate was uninformative because of its experimental design (online supplementary figure 1b).

During NMP of porcine kidneys, overall metformin clearance was higher than the creatinine clearance (P_interaction_<0.01, figure 2D). The metformin-to-creatinine clearance ratio peaked at 5.7±1.2 after 30 min of NMP and declined subsequently (linear regression equation=−0.014×min+5.4, R^2^=0.78, p<0.01; figure 2E). For the first 90 min, both metformin clearance and creatinine clearance increased over time (figure 2D). Metformin clearance decreased until the end of NMP with 48.9±19.7 mL/min/100 g (paired Student’s t-test, p=0.01), while creatinine clearance remained relatively stable (declining in total 5.0±19.7 mL/min/100 g, paired Student’s t-test, p=0.80). Non-linear curve fitting of the cross-sectional relation of metformin plasma concentration with the elimination rate using a Michaelis-Menten model showed substantial variation of the data (figure 2F, R^2^=0.49), but suggests saturation with a K_m_ of 23.0 (95% CI 10.0 to 52.3) mg/L.

### Metformin tissue level

Metformin level was measured in homogenized cortical kidney tissue of the contralateral kidney after nephrectomy, and of experimental kidneys at the end of NMP in both experiments (figure 2G). The metformin tissue levels in non-perfused kidneys of rats that received metformin pretreatment were higher than the tissue levels found after saline pretreatment (25.4±4.7 vs 0.9±0.7 mg/kg, p<0.01). Compared with controls receiving no metformin at all, metformin tissue concentration was increased in rats that received metformin pretreatment but whose kidneys were not perfused with metformin (3.0±0.8 vs 16.7±5.1 mg/kg, p=0.04). Metformin tissue level was higher in 30 mg/L perfused kidneys than kidneys perfused without metformin and was elevated even more in kidneys perfused with 300 mg/L (ANOVA, p<0.01). Pretreatment with metformin or saline did not affect metformin tissue level in the 30 mg/L (p=0.50) or 300 mg/L group (p=0.64). Porcine tissue metformin level did not differ with the 300 mg/L group in the rat study (p=0.93).

### Tubular function and renal metabolism

**Figure 3:**
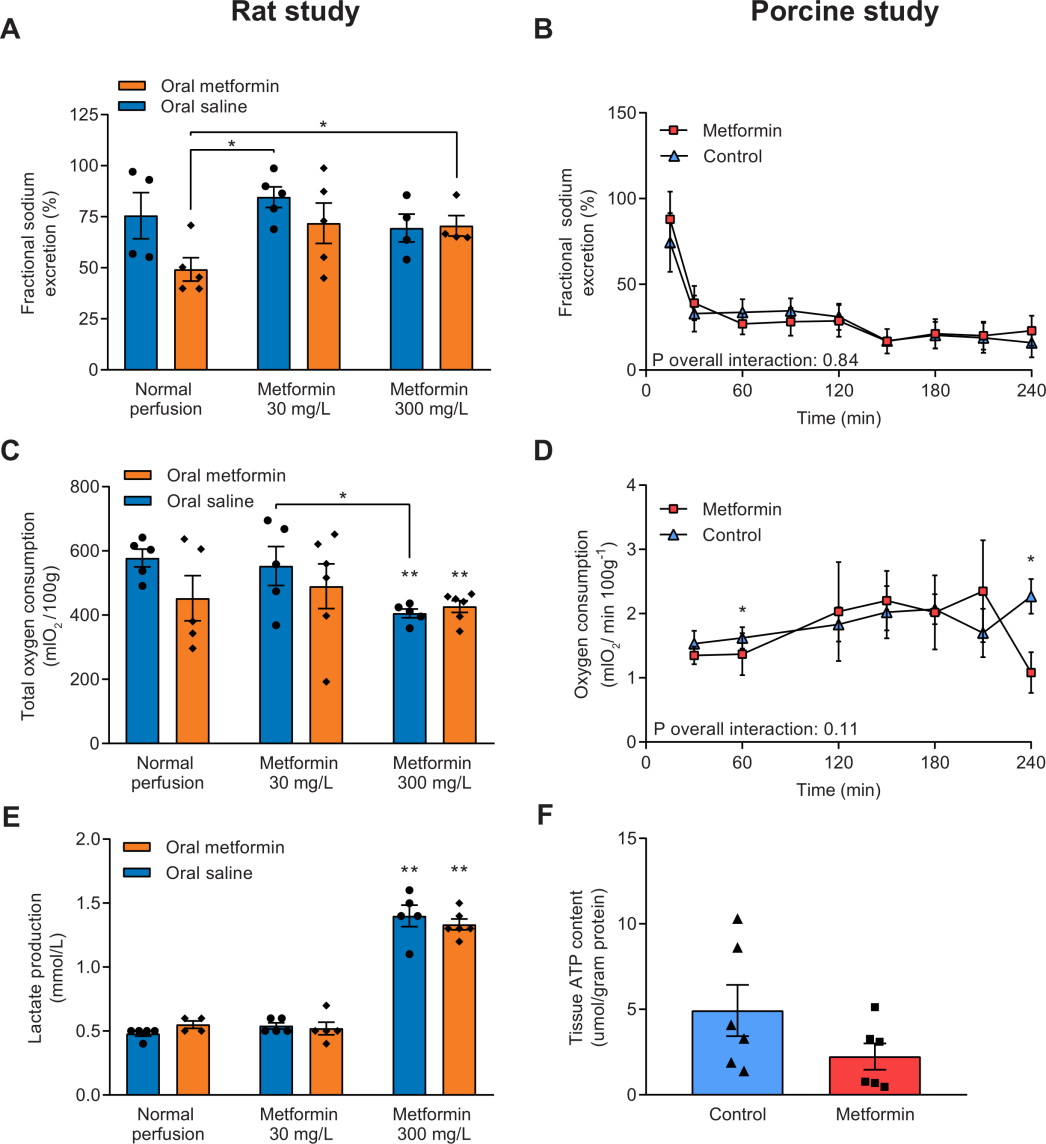
(A) Fractional sodium excretion, an indicator for tubular function, during the last 30 min of normothermic machine perfusion (NMP) in rat kidneys and (B) during NMP of porcine kidneys. Lower fractional sodium excretion corresponds with improved tubular function. (C) Total oxygen consumption of rat kidneys by calculating the area under the curve of oxygen consumption at different time points, and (D) oxygen consumption during NMP of porcine kidneys. (E) Lactate production of rat kidneys during NMP. (F) ATP content measured in cortical renal tissue at the end of NMP in porcine kidneys. Data are expressed as mean±SEM in all panels. *P<0.05. **P<0.05 vs controls (ie, saline pretreated rats whose kidneys were not perfused with metformin).

Fractional sodium excretion was calculated as an indicator for tubular function, and a lower percentage of sodium being excreted corresponds with improved tubular function. A significant interaction between treatment group and time was found for fractional sodium excretion in the rat study (P_interaction_=0.04, online supplementary figure 3a). At 60 and 90 min of NMP, rats pretreated with metformin whose kidneys were subsequently perfused without the addition of metformin had a significantly lower fractional sodium excretion compared with all other treatment groups (online supplementary figure 3, figure 3A). In the porcine kidney study, fractional sodium excretion was not different between treatment groups (P_interaction_=0.84, figure 3B).

No interaction of treatment group with time was found for oxygen consumption in the rat study (P_interaction_=0.29; online supplementary figure 3b). However, the total amount of oxygen consumed during the experiment was significantly lower in kidneys perfused with 300 mg/L metformin, irrespective of pretreatment, than controls (578±28 (controls) vs 427±18 (saline pretreatment, 300 mg/L metformin perfusion) vs 405±13 (metformin pretreatment, 300 mg/L metformin perfusion) mLO_2_/100 g, p<0.01 vs controls; figure 3C). Oxygen consumption during NMP of porcine kidneys was not affected by metformin therapy (P_interaction_=0.11; figure 3D). Also, total oxygen consumption did not differ between metformin-treated porcine kidneys and controls (296±40 vs 382±33 mLO_2_/100 g, p=0.13).

The baseline lactate level was negligible in all rat kidney treatment groups (data not shown). Compared with all other treatment groups, lactate production nearly tripled in kidneys perfused with 300 mg/L metformin, irrespective of metformin pretreatment (figure 3E). Coupled to lactate production, perfusion with 300 mg/L metformin led to increased glucose consumption compared with all other experimental groups (online supplementary figure 4). No difference in ATP content was observed between the metformin and control group in the porcine kidney study (2.2±0.8 vs 4.9±1.5 µmol/g protein, p=0.14; figure 3F). Both at the start and the end of NMP of rat kidneys, the pH in the perfusion fluid of kidneys perfused with 30 mg/L metformin approximated physiological levels, while it was decreased in otherwise treated kidneys (online supplementary figure 5a, b). The pH decreased in metformin-treated porcine kidneys, while it remained relatively stable in controls, but no statistically significant differences were found between the treatment groups (P_interaction_=0.26, online supplementary figure 5c).

### Gene expression of transporters

**Figure 4:**
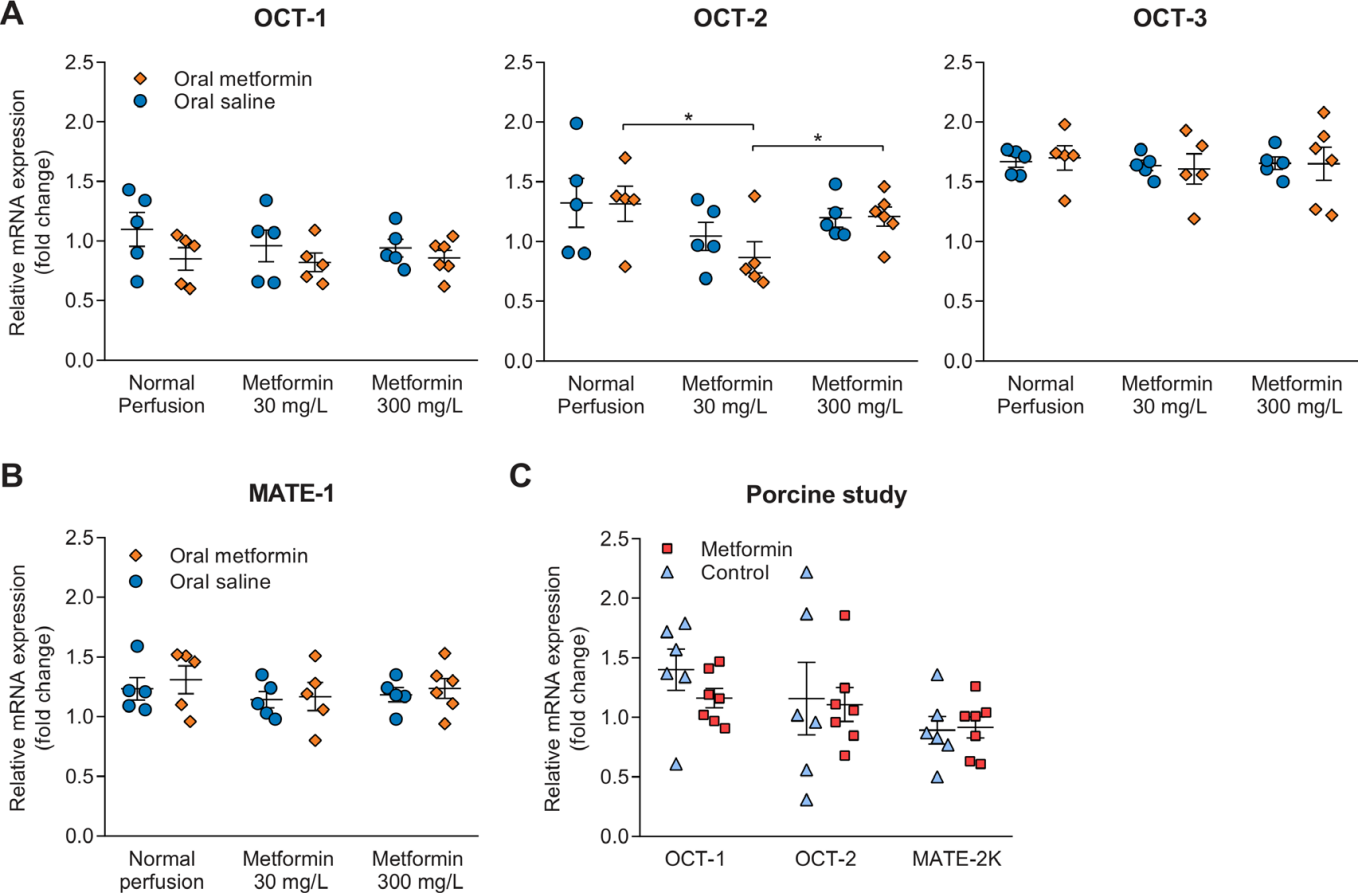
(A, B) Gene expression encoding for transporters present in the proximal tubules of the rat kidneys that are involved in basolateral uptake (OCT-2), and apical efflux of metformin, respectively (OCT-1, MATE-1 and MATE-2K). The exact role of OCT-3 in metformin transport is currently unclear. (C) Gene expression of transporters in porcine kidneys. Data are generated through real-time PCR using cortical kidney tissue obtained at the end of normothermic machine perfusion, and are expressed as mean±SEM in all panels. Primers used for this analysis are provided in online supplementary table 4. *P<0.05. MATE, multidrug and toxin extrusion transporter; OCT, organic cation transporter.

Expression of genes encoding for transporters involved in the transport of metformin was determined in both studies at the end of NMP. Gene expression of OCT-2, transporting metformin into the proximal tubule cell, in rats pretreated with metformin whose kidneys subsequently were perfused with 30 mg/L metformin was lower than gene expression in metformin pretreated rats whose kidneys were perfused without metformin or with 300 mg/L metformin, respectively (figure 4A). Expression of OCT-1 and OCT-3 was not different between treatment groups. Gene expression MATE-1, encoding for a transporter involved in the apical efflux of metformin, was not different between treatment groups in the rat study (figure 4B). In the porcine kidney study, gene expression of OCT-1, OCT-2 and MATE-2K was unaffected by metformin treatment (figure 4C).

## Discussion

In this study using two different ex vivo perfusion models, we found that secretion accounts for a substantial proportion of the total elimination of metformin. Metformin excretion was hampered at higher circulating concentrations of metformin. In contrast to our hypothesis, this can mainly be explained by the saturation of OCTs rather than a self-inhibitory effect. Interestingly, rats pretreated with oral metformin whose kidneys were perfused without the addition of metformin had the highest metformin excretion, and the highest sodium reabsorption rate (figures 2A and 3A). Although this group did not have improved fractional sodium excretion compared with controls, metformin pretreatment might improve tubular function.^23–25^ Remarkably, renal function and metabolism of these ex vivo perfused kidneys seems to be relatively resistant to metformin concentrations considered highly toxic in vivo.

Both in vivo and in vitro studies have reported that mitochondrial respiration and tissue oxygen consumption is decreased when exposed to metformin.^26–29^ Such an effect is also seen in patients treated for metformin intoxication.^30^ In our study, oxygen consumption decreased in rat kidneys perfused with 300 mg/L metformin. Possibly, the relatively short period in which the kidneys were exposed to metformin might explain the discrepancy with previous studies assessing oxygen consumption. On the other hand, acute administration of metformin to rats was also reported not to be associated with altered oxygen demand in general and, specifically, within tubules.^31^ Lactate production was increased in rat kidneys perfused with 300 mg/L metformin, indicating increased anaerobic metabolism. Because lactate production was only affected when exposed to such a high metformin concentration, other tissues than the kidney may have a considerable role in development of metformin-induced lactic acidosis as this already occurs under lower metformin concentrations. Indeed, our group reported previously that oral administration of metformin alone caused a threefold increase in lactate production during isolated liver perfusion without addition of metformin as postconditioning agent, which is in line with other studies investigating the inhibitory role of metformin on hepatic gluconeogenesis.^32–34^ Beside inhibition of lactate metabolism, lactate production by porcine skeletal muscle was increased when a high concentration of metformin was administered locally.^35^ Likewise, metformin dose-dependently caused lactate overproduction in human platelets.^36^

Beside the potential effect of metformin on its own elimination, OCTs are saturable at high concentrations as used in our study. Previously, it was reported that the apparent K_m_ ranges from 36.8 to 409.4 mg/L for OCT-2, from 29.3 to 100.7 mg/L for MATE-1 and from 135.6 to 255.7 mg/L for MATE-2K, respectively.^37^ Inhibition of MATE transporters seems to have a more important role in metformin transport and accumulation than transporters involved in influx of metformin, such as OCT-2.^38–40^ Although we assessed elimination of metformin within the whole kidney rather than a cell line expressing a single transporter, we found a K_m_ of 23 mg/L which is in line with previous reports. Moreover, the metformin tissue level of rats whose kidneys were perfused with 300 mg/L metformin was approximately a 5-fold higher than kidneys perfused with 30 mg/L metformin, while a 10-fold increase in tissue level could be expected. Assuming that metformin tissue binding is low and hence unbound concentrations in tissue should be similar to the total concentration, saturation of the transporters could be a likely explanation of our observations.

Another explanation of our findings might be that metformin, being a hydrophilic base, affects the hydrogen-ion gradient within proximal tubules. The pH in the perfusion fluid during NMP of porcine kidneys decreased over time, which is of particular interest as the transport function of metformin transporters are compromised under acidic circumstances.^7^ However, it has been previously reported that transport by OCTs is only affected when the pH was below 6.9.^41^ Studies competitively inhibiting different renal transporters report that metformin secretion can be affected by several compounds.^2 7^ However, metformin has often been investigated as a substrate and, as far as we know, it has not been reported whether increasing metformin levels affect its elimination in an ex vivo isolated kidney perfusion model. Beside passive filtration, metformin and creatinine both are transported within the proximal tubules by the same influx transporter, OCT-2.^2^ Therefore, high creatinine concentrations might competitively interact with tubular transport of metformin.^42^ However, a similar amount of creatinine was used in both models, diminishing this effect across experimental groups. When exposed to extremely high metformin concentrations, we found that indicators of renal function were relatively unaffected. Long-term metformin use of patients under normal circumstances and during acute illness was associated with mixed, but in all cases, limited effects on renal function.^43–49^

This study has important clinical implications. Metformin is currently contraindicated in patients with significant renal dysfunction because of the risk of metformin accumulation and lactic acidosis.^4^ A better understanding of the pathophysiological mechanism causing metformin-associated lactic acidosis is paramount to identify patients at risk for this severe complication of metformin therapy. We believe our findings suggest that when metformin exceeds a certain threshold level, these transporters might get overwhelmed ultimately leading to metformin-induced lactic acidosis.

Surprisingly, the therapeutic concentrations of metformin in humans remain unknown. The US Food and Drug Administration reports that 5 mg/L is the maximal level measured during controlled clinical trials which served as basis of approval for metformin, and thus this level is often considered as toxic threshold.^50^ In rats pretreated with metformin whose kidneys were not perfused metformin and during the first 90 min of normothermic perfusion of porcine kidneys, the metformin concentration in plasma remained below this level. Importantly, the metformin concentrations used in the other experimental groups in the rat study, and during the second half of NMP of porcine kidneys should be considered as massive doses which are associated with severe toxicity in both animals and humans when administered systemically.^30 36^

This study has some limitations which have to be pointed out. To increase the robustness of our findings, we applied two different study designs using parallel pretreated groups with distinct doses of metformin in the rat study and administration of increasing amounts of metformin during NMP in the porcine study. However, the concentration of metformin in perfusion fluid increased quickly, which might have affected our ability to find an effect as we did not measure metformin clearance continuously. We investigated the effects of metformin on its elimination at a functional level, using clinically relevant biomarkers that unfortunately could not provide more in-depth answers. We were unable to determine alterations in mitochondrial respiration, as that would require freshly isolated mitochondria. Furthermore, the results of the rat study were largely dependent on the comparison of rats pretreated with metformin whose kidneys subsequently were not perfused with metformin on the one hand with the other experimental groups on the other hand. In contrast, we only assessed changes over time of metformin-treated kidneys in the porcine study. Probably inherent to NMP models in general,^14–16 18^ substantial variability is apparent for all outcome parameters including metformin clearance. Because we found similar dose-dependent results across both models, we do not believe that variability in renal function hampers interpretation of our results.

In conclusion, metformin clearance was considerably higher than creatinine clearance, indicating secretion of metformin during ex vivo NMP of both rat and porcine kidneys. Metformin clearance was reduced under increasing concentrations of plasma metformin, whereas creatinine clearance and fractional sodium excretion remained relatively unaffected. This can be explained by the saturation of OCTs rather than a self-inhibitory effect, but this observation should be considered in light of short-term exposure to high levels of metformin in our study. As patients with metformin-induced lactic acidosis presumably are exposed to toxic metformin concentrations for a longer period, it remains unknown whether a self-inhibitory effect contributes to metformin accumulation in humans.
