# Supplementary material for: Increasing metformin concentrations and its excretion in both rat and porcine ex vivo normothermic kidney perfusion model
Source: BMJ Open Diabetes Res Care. 2020 Aug 17;8(1):e000816. doi: 10.1136/bmjdrc-2019-000816 (PMC7437879; doi:10.1136/bmjdrc-2019-000816)
Supplement: Supplementary data [file bmjdrc-2019-000816supp001.pdf]

Supplemental material

Increasing metformin concentrations and its excretion in both a rat and porcine ex-vivo normothermic kidney perfusion model

Rene A. Posma, Leonie H. Venema, Tobias M. Huijink, Andrie C. Westerkamp, A. Mireille A. Wessels, Nynke J. de Vries, Frank Doesburg, Jan Roggeveld, Petra J. Ottens, Daan J. Touw, Maarten W.N. Nijsten, and Henri G.D. Leuvenink

Posma RA, *et al. BMJ Open Diab Res Care* 2020; 8:e000816. doi: 10.1136/bmjdrc-2019-000816

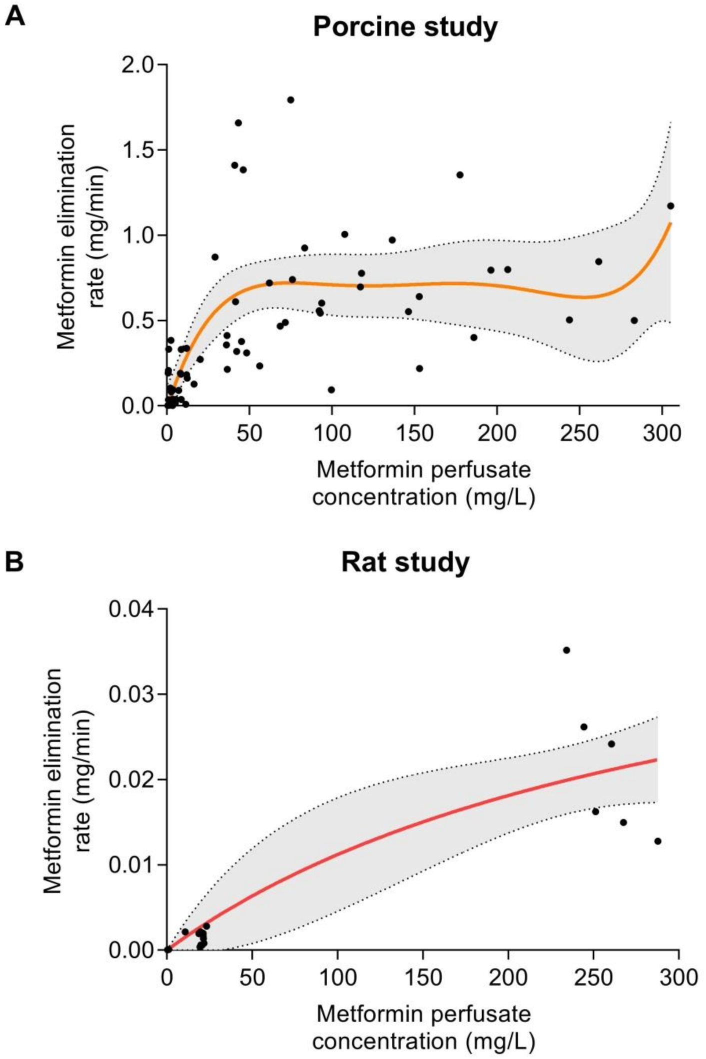

Supplementary Figure 1 – Fit with 95% confidence interval of the relation of metformin perfusate concentration with the urinary elimination rate obtained from (A) a centered fifth-order polynomial model in the porcine study and (B) a Michaelis-Menten model in the rat study.

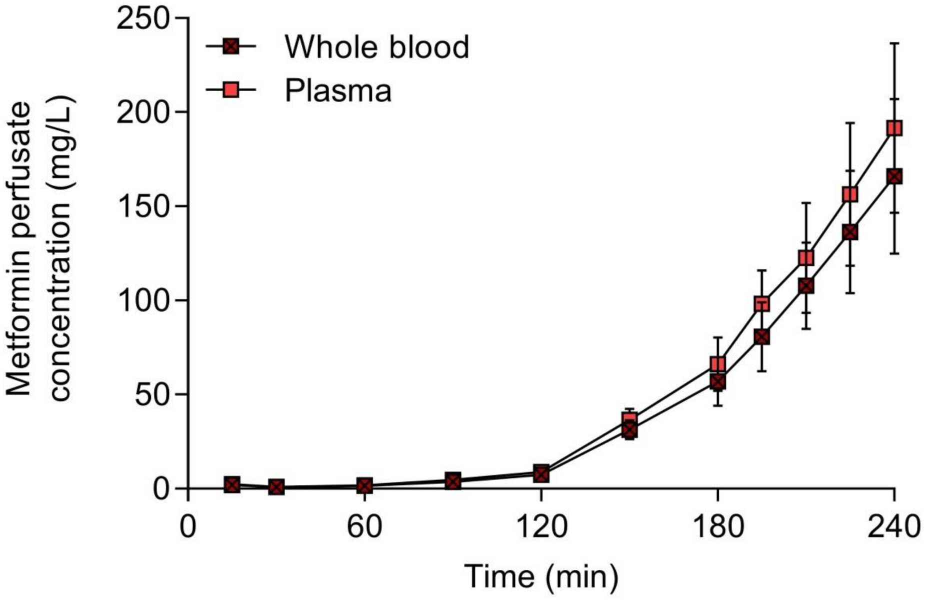

Supplemental Figure 2 – Metformin concentration in whole blood and plasma during normothermic machine perfusion of metformin-treated porcine kidneys.

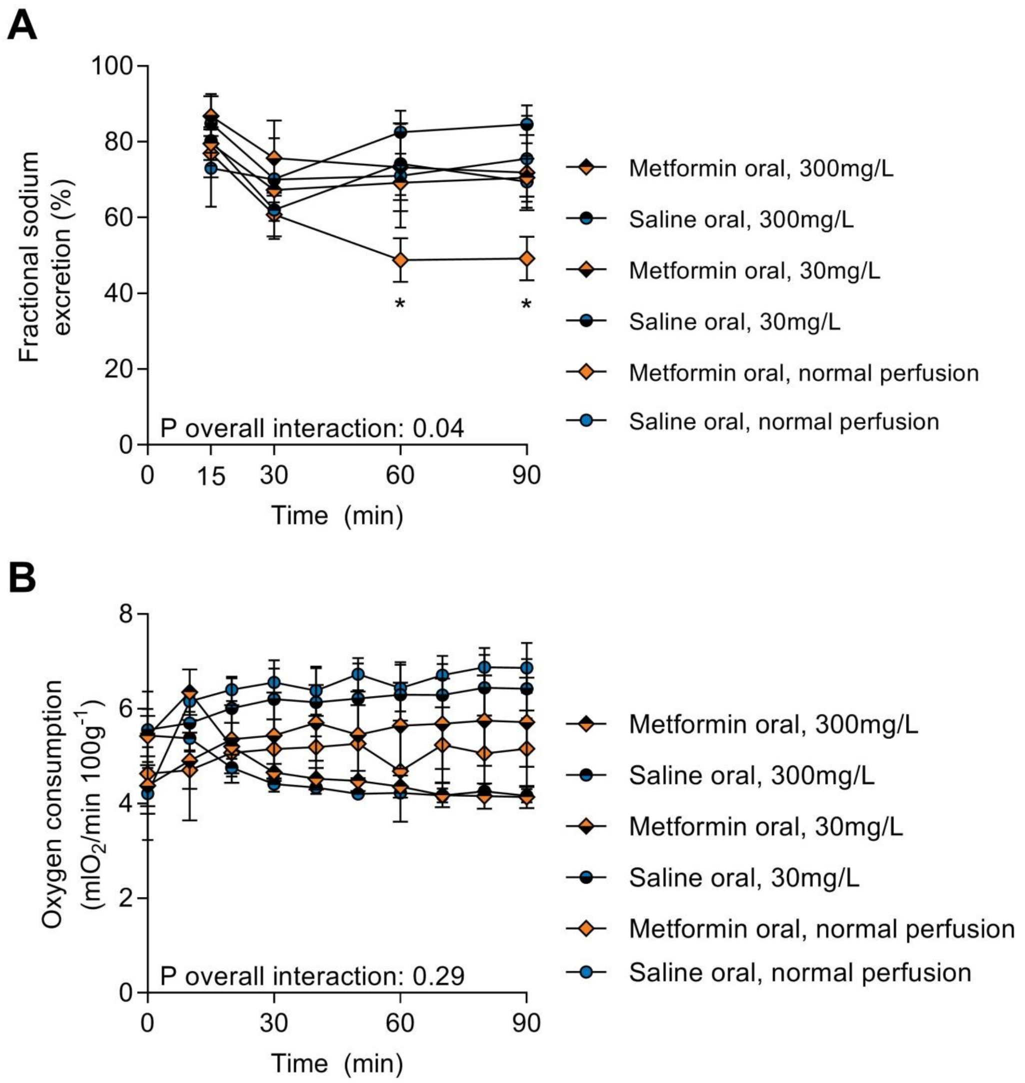

Supplemental Figure 3 – (A) Trajectories of fractional sodium excretion, an indicator for tubular function, during normothermic machine perfusion of rat kidneys. Lower fractional sodium excretion corresponds with improved tubular function. Rats were pretreated with 300 mg/kg metformin or saline twice the day before nephrectomy. * $P<0.05$ vs. all other treatment groups. (B) Trajectories of oxygen consumption, indexed for kidney weight, during normothermic machine perfusion of rat kidneys. Data are expressed as mean ± SEM.

P-values are derived from a linear mixed-effects repeated measures model with random slope and intercept on an individual level. Fixed effects were time, treatment group, and the interaction of treatment group with time. Individual kidneys were considered as a random effect. An unstructured covariance was assumed and a restricted maximum likelihood approach was used.

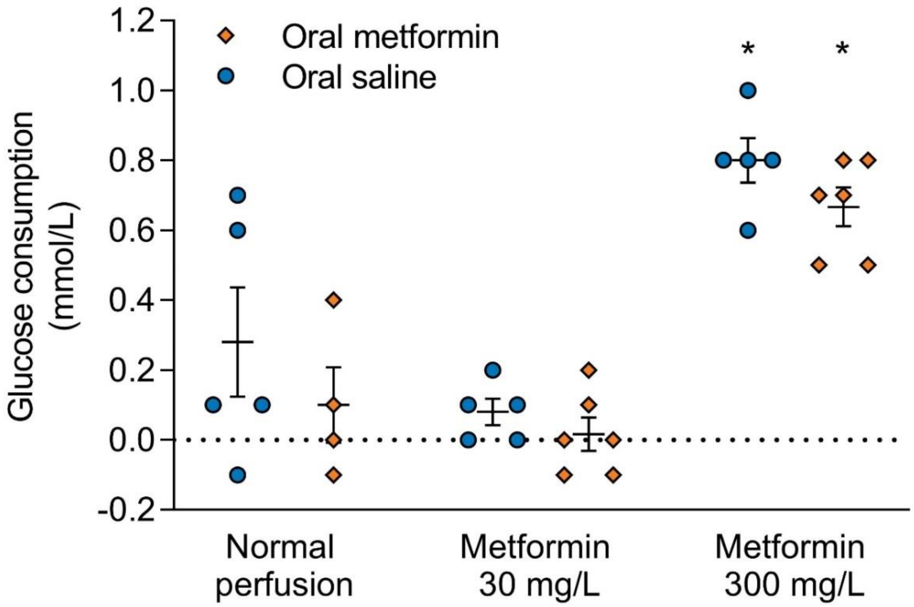

Supplemental Figure 4 – Glucose consumption of rat kidneys during 90 minutes of normothermic machine perfusion (NMP). Glucose consumption was calculated by subtracting the glucose concentration at the end of NMP from the glucose concentration at start of NMP. All glucose measurements were performed on a ABL90 FLEX blood gas analyser (Radiometer, Bronshoj, Denmark). *P≤0.03 versus all other experimental groups. Data are presented as mean ± SEM.

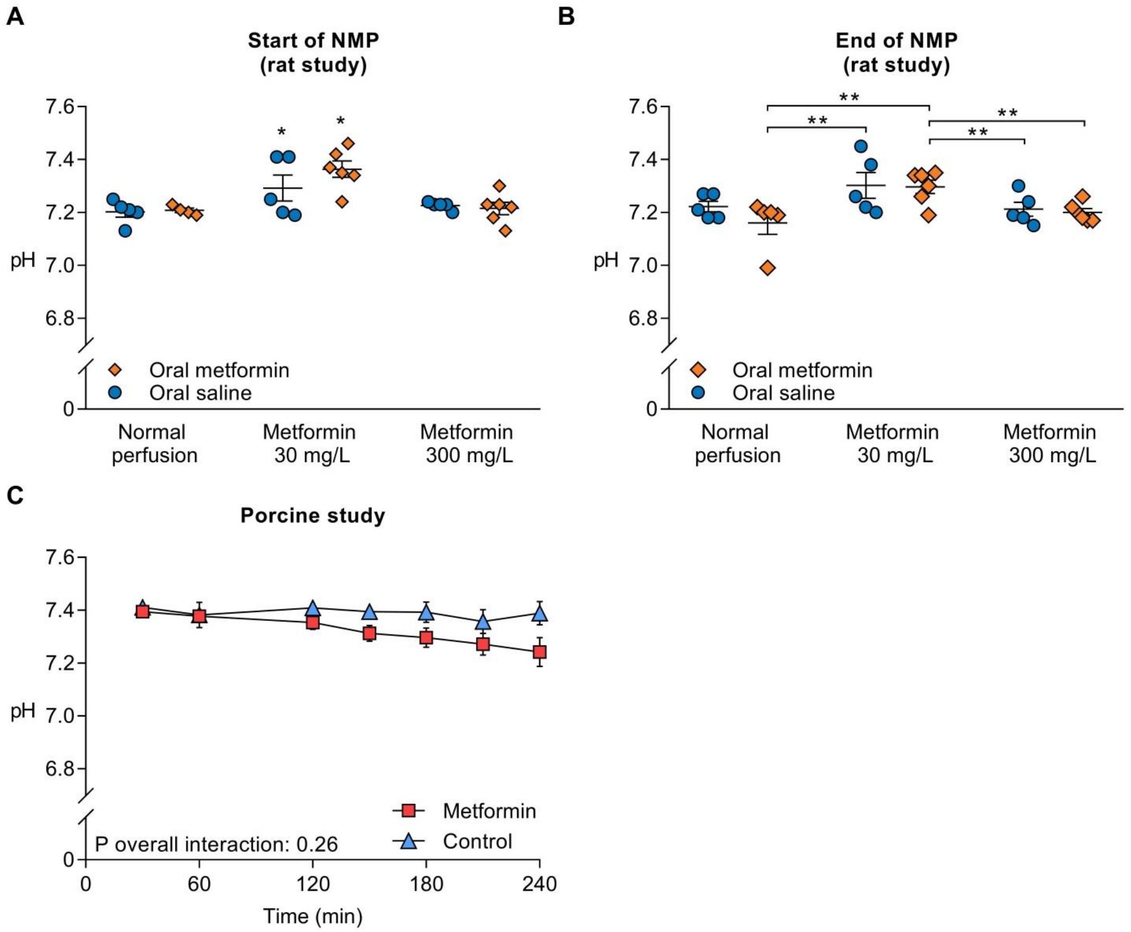

Supplemental Figure 5 – Perfusion fluid pH at the start of normothermic machine perfusion (NMP) of rat kidneys (A), and at the end of NMP of rat kidneys (B). Trajectory of pH over time during NMP of porcine kidneys (C). ***** p<0.05 vs. all other treatment groups. ** p<0.05

P-value for interaction provided for the porcine study (C) was derived from a linear mixed-effects repeated measures model with random slope and intercept. Fixed effects were time, treatment group, and the interaction of treatment group and time. Individual kidneys were considered as a random effect. The covariance matrix of residuals used in all models was unstructured. A restricted maximum likelihood approach was used.

Posma RA, *et al. BMJ Open Diab Res Care* 2020; 8:e000816. doi: 10.1136/bmjdrc-2019-000816

Supplemental Table 1 - Compounds of the perfusion fluid used in the porcine study.

**Material**	**Amount**	**Manufacturer**
Autologous leukocyte filtered blood	500 ml	Not applicable
Ringer's lactate solution	300 ml	Baxter, Utrecht, The Netherlands
Glucose 5% solution	10 ml	Baxter, Utrecht, The Netherlands
Amoxicillin-clavulanate	1000mg/200mg	Sandoz, Almere, The Netherlands
Dexamethasone	6 mg	B. Braun, Melsungen, Germany
Sodium bicarbonate 8.4%	10 ml	B. Braun, Melsungen, Germany
Creatinine	90 mg	Sigma-Aldrich, St. Louis, MO
Mannitol	6 mg	Sigma-Aldrich, St. Louis, MO
Sodiumnitroprusside	2 mg	Sigma-Aldrich, St. Louis, MO
1,1-dimethylbiguanide hydrochloride	3,3 mg	Sigma-Aldrich, St. Louis, MO
**Continuous infusion 1***		
Glucose 5%	5 ml/h	Baxter, Utrecht, The Netherlands
**Continuous infusion 2**	**20 ml/h**	
Aminoplasmal 10%	82 ml	B. Braun, Melsungen, Germany
Insulin aspart (Novorapid)	1 ml (100 IU/ml)	Novo Nordisk, Alphen a/d Rijn, The Netherlands
Sodiumbicarbonate 8.4%	2,5 ml	B. Braun, Melsungen, Germany
**Continuous infusion 3**	**Speed according to prespecified schedule**	
1,1-dimethylbiguanide hydrochloride	0.7 gram	Sigma-Aldrich, St. Louis, MO
Ringer's lactate solution	35 ml	Baxter, Utrecht, The Netherlands

* Speed adjusted to maintain a glucose level between 7.0 mmol/L and 8.0 mmol/L

Posma RA, *et al. BMJ Open Diab Res Care* 2020; 8:e000816. doi: 10.1136/bmjdrc-2019-000816

Supplemental Table 2 – Prespecified metformin infusion speed schedule.

**Time (mins)**	**mg/h**	**Cumulative amount of metformin administered (mg)**
0	15	0
30	20	7.5
60	40	17.5
90	70	37.5
120	170	72.5
150	210	157.5
180	260	262.5
210	310	392.5
240	Stop infusion	547.5

Posma RA, *et al. BMJ Open Diab Res Care* 2020; 8:e000816. doi: 10.1136/bmjdrc-2019-000816

Supplemental Table 3 – Primers used for real-time PCR.

Gene	Primers		Amplicon size (bp)
Rat study			
β-actin	GGAAATCGTGCGTGACATTAAA	GCGGCAGTGGCCATCTC	74
OCT-1	AGGCAAGTCCTCAAGTACCTGACAG	CCAAGTGGCAAGTCCTCCTTT	77
OCT-2	CCATTGCATGCTTGGGTAGAA	ACAAGGACACCAAGATTCCTGATGTA	105
OCT-3	TAACGTGTGGGCTCGAGTAGGA	ACATTAGGGATGAAGGGCTGC	80
MATE-1	TGGTGGGTGGGATTTTAGTGAG	AGCACCTGCATTGCTGGATTA	76
Porcine study			
β-actin	TCTGCGCAAGTTAGGTTTTGTC	CGTCCACCGCAAATGCTT	92
OCT-1	GCGAGGTCGACGGAGTTATTATCT	TGGGAGCAGGCAAGAAGGA	82
OCT-2	TCGCAAAGCACATGCAATTC	GGCAGACACACTGGTATACAGAACA	93
MATE-2K	AGCATCCTGAGGAATAAACTGGG	GATATAAAGCGGCAGGGCCTT	86

Posma RA, *et al.* *BMJ Open Diab Res Care* 2020; 8:e000816. doi: 10.1136/bmjdrc-2019-000816
